# Manual of the Diseases of the Eye

**Published:** 1905-11

**Authors:** 


					﻿Manual of the Diseases of the Eye.—By Charles H. May, M.
D., Ophthalmic Suregon to the City Hospitals, Randall’s Island,
New York, etc. Fourth edition revised,- with 860 original il-
lustrations including 21 plates and 60 colored figures. Four
hundred pages. Price $2. William Wood & Co., 51 Fifth Ave-
nue, New York.
May on The Eye has taken a strong hold on the profession.
Several editions have been exhausted since it was first published
a short while ago, and the demand is still for more. It is ex-
cellent.—D.
				

